# Efficacy of a novel topical combination of esafoxolaner, eprinomectin and praziquantel against *Echinococcus multilocularis* infections in cats

**DOI:** 10.1051/parasite/2021025

**Published:** 2021-04-02

**Authors:** Joe Prullage, Dwight Bowman, Michael Ulrich, Eric Tielemans

**Affiliations:** 1 Boehringer-Ingelheim Animal Health, Missouri Research Center 6498 Jade Rd. Fulton 65251 MO USA; 2 Cornell University, College of Veterinary Medicine Ithaca 14850 NY USA; 3 Cheri Hill Kennel and Supply Inc. 17190 Polk Rd. Stanwood 49346 MI USA; 4 Boehringer-Ingelheim Animal Health 29 Avenue Tony Garnier 69007 Lyon France

**Keywords:** Cat, Praziquantel, Esafoxolaner, Eprinomectin, Cestode, Efficacy, *Echinococcus multiloculari*s

## Abstract

NexGard^®^ Combo, a novel topical endectoparasiticide formulation for cats combining esafoxolaner, eprinomectin and praziquantel, for the treatment of internal and external parasite infestations, including arthropods, nematodes and cestodes, was tested for efficacy against induced infections of *Echinococcus multilocularis* in cats, in two experimental studies. The two studies were performed in the United States with the same *E. multilocularis* isolate sourced locally. In each study, 20 cats were inoculated intra-gastrically with ~30,000 *E. multilocularis* protoscoleces three weeks before treatment, then ten cats were randomly allocated to a placebo control group or to the novel formulation treated group. Inoculated cats were treated topically once at the minimum recommended dose of the novel formulation, or with an identical volume of placebo. One week after treatment, cats were humanely euthanized for parasite recovery and count. The efficacy calculation was based on comparison of number of scoleces found in the control group and the novel formulation group. In the two control groups, *E. multilocularis* scoleces were found in five (range: 30–1025) and eight (range 2–345) cats, the geometric means inclusive of the ten cats per group were 8.9 and 28.8, respectively. In the two novel formulation-treated groups, none of the cats harbored any *E. multilocularis* scoleces, demonstrating 100% efficacy.

## Abstract (fr)

NexGard^®^ Combo, une nouvelle formulation d’endectoparasiticide topique pour chats combinant l’esafoxolaner, l’éprinomectine et le praziquantel pour le traitement des infestations parasitaires internes et externes, y compris les arthropodes, les nématodes et les cestodes, a été testé pour son efficacité contre les infections induites par *Echinococcus multilocularis* chez les chats, dans deux études expérimentales. Les deux études ont été réalisées aux États-Unis avec le même isolat d’*E. multilocularis* d’origine locale. Dans chaque étude, vingt chats ont été inoculés par voie intra-gastrique avec ~ 30 000 protoscolex d’*E. multilocularis* trois semaines avant le traitement, puis dix chats ont été répartis au hasard dans un groupe témoin placebo ou dans le groupe traité par la nouvelle formulation. Les chats inoculés ont été traités une fois par voie topique à la dose minimale recommandée de la nouvelle formulation, ou avec un volume identique de placebo. Une semaine après le traitement, les chats ont été euthanasiés sans cruauté pour la récupération et le dénombrement des parasites. Le calcul de l’efficacité était basé sur la comparaison du nombre de scolex trouvés dans le groupe témoin et le groupe de formulation nouvelle. Dans les deux groupes témoins, des scolex d’*E. multilocularis* ont été trouvées chez cinq (extrêmes: 30 – 1025) et huit (extrêmes: 2 – 345) chats, les moyennes géométriques comprenant les dix chats par groupe étaient respectivement de 8,9 et 28,8. Dans les deux groupes traités par formulation nouvelle, aucun des chats ne portait de scolex d’*E. multilocularis*, démontrant une efficacité de 100 %.

## Introduction

*Echinococcus multilocularis* is endemic in the cold and temperate regions of the Northern Hemisphere, e.g. in Central and Eastern Europe and in various regions of North America and Asia [15, 16, 19, 23, 28, 30, 31, 33, 44]. Increasing prevalences of the parasite and spread to new territories and also to urban areas in endemic countries such as Switzerland, Germany, Denmark and regions of China and Alaska, have been reported over the last two decades [10, 11, 13, 18, 34, 35]. The United Kingdom is an exception, where there is no evidence of *E. multilocularis* presence in any wild or domestic species, which is amongst the justifications for the currently enforced pet travel scheme [27].

The sylvatic cycle of *E. multilocularis,* involving wild canids such as foxes (considered the main definitive host), coyotes, badgers or raccoons dominates the epidemiology of this cestode, with up to 70% prevalences in some areas [8, 24, 34]; however, it can occasionally involve domestic dogs and cats exposed to infected intermediate hosts. Infection is usually asymptomatic in definitive hosts, which may harbor several hundred adult cestodes. A high number of eggs are shed in the feces of the definitive host and may remain infective for years in the environment, in cool and humid conditions [6]. Eggs are ingested by intermediate hosts, i.e. various rodents such as voles, muskrats, lemmings, hamsters, gerbils and related species, which develop alveolar echinococcosis. The multi-alveolar larva progressively infiltrates the infected organ, usually the hepatic parenchyma, in 1–3 months. The metacestode larva is formed of a high number of vesicles, each containing high numbers of protoscoleces, the infective stage for the definitive hosts. When infected rodents are preyed and ingested by the definitive host, protoscoleces grow into adult *E. multilocularis* in the small intestine, and egg production can begin as early as 25 days after ingestion. Eggs may also be ingested by aberrant hosts, such as pigs, horses, monkeys and humans, causing alveolar echinococcosis, one of the most lethal helminth infections in humans [3, 4, 8, 12].

The susceptibility of dogs to *E. multilocularis* infection is comparable to that of foxes, in terms of pre-patent period and numbers of eggs produced, however with a lower prevalence, probably because of their feeding habits resulting in lower exposure to infected intermediate hosts [24, 25]. The importance of cats in the epidemiology of *E. multilocularis* is not yet fully understood. Experimental *E. multilocularis* infection studies in cats, in comparison to foxes or dogs have shown a shorter pre-patent period, lower number of adult cestodes in the intestine, and lower number of eggs produced [24, 36, 37, 39]. However recent studies suggest that cats might be underestimated in their role as potential hosts and as reservoirs impacting environmental and human exposure, especially when living in highly endemic areas [25, 26]. This is supported by recent studies that identified a significant level of cat feces contaminated with *E. multilocularis* eggs in kitchen gardens, which may cause human exposure [2, 29].

Human alveolar echinococcosis (AE), a severe and life-threatening foodborne parasitic disease is caused by larval *E. multilocularis* [9, 12, 17, 42]. Usually diagnosed incidentally on investigation of abdominal or general signs, it often requires surgery and long-lasting drug therapy, and the prognosis is poor at the late stage [5]. AE is common in some rural areas of China [43, 45]. In Central Europe, annual incidences of 0.03–1.2 cases per 100,000 inhabitants have been reported [8]. While the other human echinococcosis, cystic echinococcosis (CE) caused by *Echinococcus granulosus,* has been significantly reduced by application of prevention and control measures such as dog deworming, meat inspection and human population surveillance, AE has increased in continental Europe and is a major public health concern in Eurasia [9, 43].

NexGard^®^ Combo, a novel topical endectoparasite formulation for cats combines esafoxolaner, an isoxazoline compound with insecticidal and acaricidal properties; eprinomectin, an avermectin of the macrocyclic lactone class, active against nematodes; and praziquantel, a pyrazino-isoquinoline derivative anthelminthic that acts specifically on cestodes and trematodes. Praziquantel has been used for decades in several animal species and by several routes of administration and is an established compound with proven safety and efficacy against cestodes, including *E. multilocularis* [1, 7, 22, 32]. Broadline™, a marketed feline topical product, containing praziquantel in identical concentration, volume and dosage as in the novel formulation, was demonstrated 100% efficacious against *E. multilocularis*-induced infections in cats [38].

This manuscript describes two experimental studies designed to evaluate the efficacy of NexGard^®^ Combo against *E. multilocularis-*induced infections in cats.

## Materials and methods

### Ethics

The study protocols were reviewed and approved by the Sponsor’s and local Institutional Animal Care and Use Committees. Cats were managed and handled with due regard for their wellbeing.

### Study designs

The two study designs were in accordance with the International Cooperation on Harmonisation of Technical Requirements for Registration of Veterinary Medicinal Products VICH GL7, “Efficacy of Anthelmintics: General Requirements” [40] and VICH GL20 “Efficacy of Anthelmintics: Specific Recommendations for Felines” [41], and the “World Association for the Advancement of Veterinary Parasitology (WAAVP) guidelines for evaluating the efficacy of anthelmintics for dogs and cats” [20]; and the studies were conducted in compliance with VICH GL9 entitled Good Clinical Practice.

The two studies were conducted in the same facility in the United States and had an identical design. The first study was performed in September 2017, the second in February 2018.

The studies were conducted under a negative-controlled and randomized design, based on bodyweight. Personnel involved with evaluation of efficacy and safety were unaware as to treatment assignments. *Echinococcus multilocularis-*inoculated cats were treated with the novel formulation, at the minimum effective dose or with a placebo. The efficacy assessments were based on comparison of *E. multilocularis* scolex counts in the placebo- and novel formulation-treated cats.

### Animals, husbandry and health

Twenty purpose-bred healthy Domestic Short-hair cats, sourced from the same supplier were included in each study and are described in Table 1.

**Table 1:** Characteristics of experimental animals.

Study	Sex	Age (Day 0)	Bodyweight
Study #1	10 males, 10 females	12–13 months	2.4–5.4 kg (Day −3)
Study #2	10 males, 10 females	6 months	2.3–3.9 kg (Day −2)

None of the cats had been treated with an endoparasiticide compound within 3 months of study start. Cats were single housed to avoid inter-animal treatment contamination, in a controlled environment. Cats were identified individually with ear tattoos. Cats were examined by a veterinarian for suitability before *E. multilocularis* inoculation, were observed at least once daily for general health for the whole study, and were observed hourly four times after treatment application, to monitor any health abnormality or reaction to treatment.

### Experimental schedule

Due to the zoonotic potential of the parasite, the inoculation schedule was designed so that *E. multilocularis* would, as much as possible remain immature, to minimize shedding of infective eggs by the cats.

Cats were acclimated to study conditions from Day −30, inoculated with *E. multilocularis* on Day −21, weighed and randomized to treatment group on Day −2, treated on Day 0, and necropsied on Day 7 or 8 for *E. multilocularis* scoleces collection and count.

### *Echinococcus multilocularis* isolate

Both studies used the same *E. multilocularis* isolate obtained from foxes in Montana, USA. Cotton rats (*Sigmodon hispidus*) were orally infected using proglottids obtained from the foxes. When the multilocular larva had matured, 32 weeks after inoculation, the protoscoleces were collected from the cotton rats. The processed material was suspended in a storage solution until use.

### Induced *E. multilocularis* infection

Cats were fasted overnight before oral inoculation with *E. multilocularis* protoscoleces on Day −21. Each cat was sedated then inoculated intragastrically via an esophageal tube with an aliquot containing approximately 30,000 *E. multilocularis* protoscoleces drawn from the same bulk solution. Vitality of protoscoleces had been verified by microscopic examination shortly before the inoculation and was based on motility.

### Treatment

Cats were treated once on Day 0. The treatment was applied topically on the skin, after parting the hair, on one spot in the midline of the neck between the base of the skull and the shoulder blades. Cats assigned to the placebo control group were treated with mineral oil at 0.12 mL/kg, cats assigned to the novel formulation treated group were applied the product at the minimum recommended dose of 0.12 mL/kg, delivering 1.44 mg/kg esafoxolaner, 0.48 mg/kg eprinomectin, and 10.0 mg/kg praziquantel.

### *Echinococcus multilocularis* counts

On Days 7 or 8, cats were humanely euthanized. For each cat, the stomach and the small and large intestines were opened along their length, the content was collected, the surface of the small intestine scraped, and the large intestine was passed through a gloved thumb and forefinger. The collected material and scrapings were washed through appropriate sieves and placed in a defined volume in a saline solution. A 20% aliquot was transferred in small amounts to a gridded Petri dish, and examined using a dissecting microscope for identification and count of *E. multilocularis*. If fewer than 6 scoleces were found, the total contents of the sample from the small intestine were examined the same way as for the 20% aliquot. If 6 or more scoleces were found in the 20% aliquot, the number was multiplied by 5 to define the total *E. multilocularis* number.

### Statistical analysis

In each study, counts of *E. multilocularis* scoleces were transformed to the natural logarithm of (count + 1) for calculation of geometric means for each group. Efficacy was determined by calculating the percent efficacy as 100 × ([*C* − *T*)/*C*], where *C* was the geometric mean count among placebo controls, and *T* was the geometric mean count among the animals treated with the novel formulation. To compare the geometric means of the two groups, the mean of the log-counts of the novel formulation treated group was compared to the mean of the log-counts of the placebo control group using an F-test adjusted for the allocation blocks used to randomize the animals to the treatment groups. The mixed procedure in SAS^®^ version 9.1.3 was used for the analyses, with the treatment groups listed as a fixed effect and the allocation blocks listed as a random effect. All testings used a two-sided significance level *α* = 0.05.

## Results

The efficacy results are summarized in Table 2.

**Table 2:** Efficacy of the novel formulation against induced *E. multilocularis* infections in cats.

	*E. multilocularis* counts				Efficacy^c^ (%)	*p*-value^d^
	Placebo group		Novel formulation group			
	NI/NG^a^	GM^b^ (range)	NI/NG^a^	GM^b^ (range)		
Study #1	5/10	8.9 (0–1025)	0/10	0.0 (0)	100.0	0.0123
Study #2	8/10	28.8 (0–345)	0/10	0.0 (0)	100.0	0.0002

a NI/NG: number of cats infected/number of cats in the group.

b GM = geometric mean.

c Percent efficacy = ([*C* – *T*]/*C*) × 100, where *T* and *C* were geometric means of the placebo and the novel formulation groups, respectively.

d *p*-value = two-sided probability value from analysis of variance on log-counts of placebo and novel formulation groups.

In Study #1, 30–1025 *E. multilocularis* scoleces were recovered from 5 placebo control cats and no scoleces were found in the other 5 cats of the group, the geometric mean of the group inclusive of the ten cats was 8.9. No scoleces were found in the novel formulation-treated group, demonstrating 100% efficacy and a significant difference between the two groups (*p* = 0.0123).

In Study #2, 2–345 *E. multilocularis* scoleces were recovered from 8 placebo control cats and no scoleces were found in the other 2 cats of the group, the geometric mean of the group inclusive of the ten cats was 28.8. No scoleces were found in the novel formulation-treated group, demonstrating 100% efficacy and a significant difference between the two groups (*p* = 0.0002).

The establishment rates of the *E. multilocularis* isolate, i.e. mean number of *E. multilocularis* scolices per number of protoscoleces inoculated in the placebo groups were 0.42% in Study #1 and 0.34% in Study #2.

In both studies, no adverse reactions related to the treatment were observed, indicating a high level of tolerance to the novel formulation.

## Discussion

The results of these two studies provide strong evidence of the high efficacy of NexGard^®^ Combo for the treatment of *E. multilocularis* infections in cats. The establishment rates in the placebo groups in these two studies (0.42 and 0.34%) add to the body of evidence that cats have low susceptibility to *E. multilocularis* infection, in comparison to foxes or dogs [14, 24, 36]. Nevertheless, in these two studies and in five previous studies evaluating experimental infections of *E. multilocularis* in cats, very high individual inconsistency of infection was observed, as the control groups always had some cats with zero worms, some with very few worms, and others with several hundred or thousands of worms [1, 22, 24, 38]. This high individual variability could not be explained with the available data; all studies used healthy DSH/DLH cats from licensed breeders’ outbred colonies, cats were 3–12 months old (six studies), no sex effect was seen (four studies), and the methodology of experimental infection used in the seven studies was comparable.

Several factors should be considered in highlighting the potential role of cats as a source of infection for humans: the increasing number of reports of infections in cats, individual variability in harboring scoleces, the close relationship between humans and cats, the reports of increasing prevalence of the parasite, the spread to urban areas and to new countries, and the predatory behavior of cats with outdoor access. These factors provide strong support that cats should be treated on a regular basis, especially when they are located in endemic areas and have outdoor access.

The results of these two studies were not surprising, praziquantel had already been demonstrated efficacious against *E. multilocularis* in cats with other topical products [7, 22], including Broadline™, 100% efficacious against two *E. multilocularis* isolates from Europe [38], in which the praziquantel dosage, volume and concentration are identical, and in view of the plasma profile of praziquantel, recently demonstrated to be similar following one application of Broadline™ or of NexGard^®^ Combo [21].

NexGard^®^ Combo offers an efficacious, safe and easy to use solution for the treatment of cats with infections of *E. multilocularis* and provides an excellent solution to mitigate the risk of transmission of this zoonotic parasite to humans.

## Competing interest

The work reported herein was funded by Boehringer-Ingelheim Animal Health. The authors are current employees of Boehringer-Ingelheim Animal Health or external organizations. Other than that, the authors declare no conflict of interest. This document is provided for scientific purposes only. Any reference to a brand or trademark herein is for information purposes only and is not intended for any commercial purposes or to dilute the rights of the respective owners of the brand(s) or trademark(s). NexGard^®^ is a registered trademark of the Boehringer-Ingelheim Group.
